# Association grossesse et pseudomyxome péritonéal secondaire à une tumeur mucineuse borderline de l’ovaire: à propos d’un cas et revue de la littérature

**Published:** 2012-10-25

**Authors:** Sofia Jayi, Hind Fatemi, Hakima Bouguern, Hikmat Chaara, My Abdelilah Melhouf

**Affiliations:** 1 Service de Gynéco-Obstétrique 2, CHU Hassan II, Fès, Maroc; 2 Service d’anatomopathologie, CHU Hassan II, Fès, Maroc

**Keywords:** Grossesse, tumeur mucineuse, ovaire, borderline pseudomyxome, diagnostic, traitement, pronostic, Pregnancy, mucinous tumor, ovary, borderline pseudomyxoma, diagnosis, treatment, prognosis

## Abstract

Les tumeurs mucineuses de l’ovaire représentent 20% des tumeurs épithéliales. La forme borderline en est une entité particulière et est de survenue rare particulièrement au cours de la grossesse (1/10 000 à 1/50 000). Nous rapportons le cas d’une patiente de 35 ans G4P3, présentant une grossesse de 22SA associée à une tumeur ovarienne droite gélatineuse, rompue avec implants péritonéaux, dont l’examen extemporané de l’annexectomie a trouvé une tumeur mucineuse au minimum borderline. Une chirurgie radicale a été faite avec à l’étude histologique définitive: une tumeur ovarienne mucineuse borderline avec tératome mature et pseudomyxome péritonéal. A travers ce cas rare et à la lumière d’une revue de la littérature nous insistons sur les caractéristiques épidémiologiques diagnostiques, thérapeutiques et pronostiques de cette rare entité tout en précisant les particularités de son association avec la grossesse.

## Introduction

es tumeurs mucineuses de l’ovaire font partie des tumeurs épithéliales ovariennes. Elles représentent environ 20% des cas. Comme pour toutes les tumeurs épithéliales ovariennes on distingue: Les tumeurs mucineuses borderlines qui représentent une entité particulière. Ces tumeurs sont souvent secondaires à une origine digestive et particulièrement appendiculaire avec un pseudomyxome péritonéal dans 33% des cas. L’association à la grossesse d’une tumeur mucineuse borderline de l’ovaire, compliquée d’un pseudomyxome péritonéal, reste un événement rare, dont la prise en charge peut dans certains cas être inadaptée. Notre travail a pour but de guider le praticien dans la prise en charge de cette entité particulière, sur la base d’une revue de la littérature.

## Patient et observation

Mme M.A. 4ème geste 3ème pare âgée de 35 ans, sans antécédents particuliers, cette grossesse non suivie est estimée à 5 mois avec notion de douleur abdominopelvienne à type de pesanteur évoluant depuis 20 jours.

**Figure 1:**
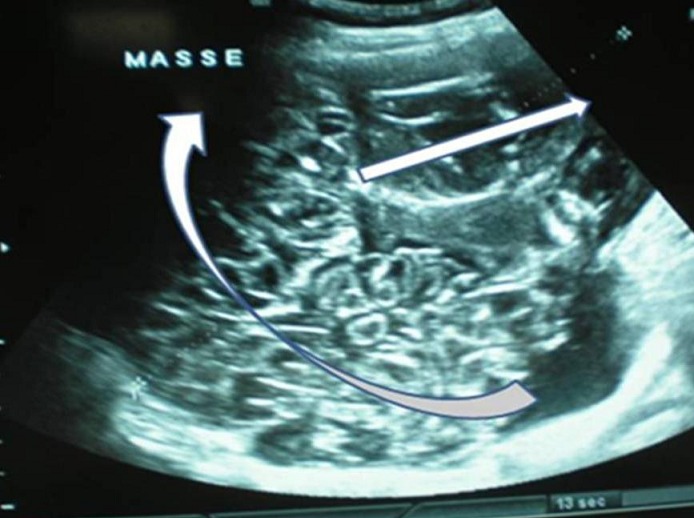
Double composante à l’échographie: aspect hypoéchogéne hétérogène (flèche droite); aspect multiloculaire avec cloisons et raccordements géométriques (flèche courbée)

**Figure 2:**
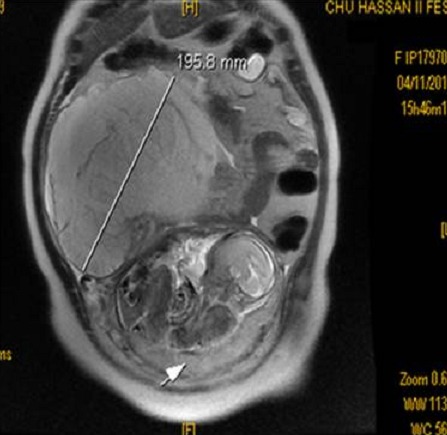
Coupe frontale à l’IRM montrant la masse abdomnino-pelvienne latéralisée à droite mesurant 19,5 cm de grand axe et la grossesse intra-utérine (flèche)

**Figure 3:**
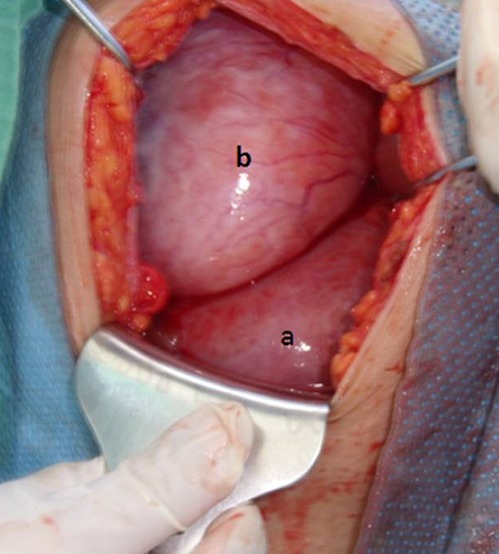
a: utérus gravide; b: pole inféro-interne de la masse ovarienne

**Figure 4:**
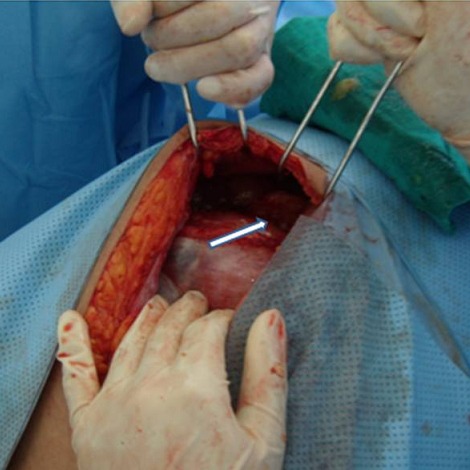
Pole supérieur de la masse, rompu avec issue de gélatine (flèche)

L’examen clinique à l’admission trouve une masse abdominopelvienne latéralisée à droite, arrivant jusqu’au flanc, mal limitée, légèrement sensible, et un utérus arrivant à l’ombilic. l’échographie trouve une grossesse monofoetale évolutive dont la biométrie est à 22 semaines d’aménorrhées, avec une image latéro-utérine droite hétérogène faite de 2 composantes: l’une échogène ayant un aspect cérébroide et l’autre hypo-échogène hétérogène le tout mesurant 17,4 cm de grand axe, sans épanchement visible en intrapéritonéal, le tout évoquant une tumeur ovarienne, mais vue l’absence de vascularisation évidente au doppler un kyste ovarien hémorragique ou un hématome sont aussi évoqués (Figure 1). L’IRM trouve un processus grossièrement arrondi à contenu majoritairement liquidien contenant de multiples cloisons fines et renfermant dans son pole inferieur une formation à 3 composantes: graisseuse, calcique et hémorragique, le tout évoquant un tératome ovarien probablement immature (Figure 2) d’où la réalisation d’une laparotomie exploratrice; laquelle a objectivé une tumeur ovarienne droite de 19 cm de grand axe (Figure 3) dont la paroi postéro-supérieure est rompue avec issu de gélatine et présence de foyers gélatineux adhérents à la gouttière pariéto-colique droite (pseudomyxome péritonéal) (Figure 4) alors que le reste de l’exploration est sans particularité. L’étude extemporanée de l’annexectomie droite trouve une tumeur mucineuse au minimum borderline. La décision de réaliser une hystérectomie totale avec annexectomie gauche, curage iliaque, appendicectomie et ommentectomie sous mésocolique et biopsies péritonéales, est prise; vu que la patiente a 3 enfants et qu’elle avait refusé en préopératoire toute reprise opératoire secondaire.

**Figure 5:**
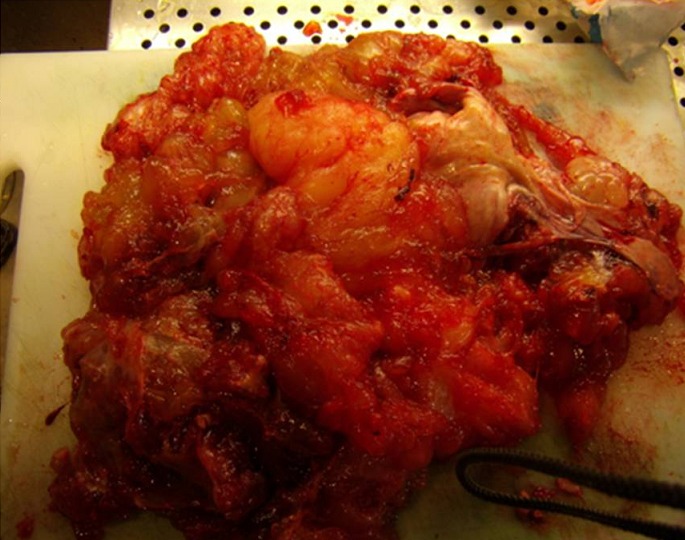
Aspect macroscopique de la tumeur après son ouverture

L’étude anatomopathologique définitive trouve - macroscopiquement - un aspect hétérogène à la coupe (Figure 5), et à l’histologie une tumeur mucineuse borderline de l’ovaire droit avec présence de tissus musculaire, adipeux, cutané, respiratoire réalisant un tératome mature (Figure 6). En outre, l’étude a objectivé la présence de pseudomyxome péritonéal sur l’omentectomie et le prélèvement de la gouttière pariétocolique droite, alors que l’utérus, le placenta, l’appendice, la gouttière pariétocolique gauche étaient sans anomalies, et le curage a ramené 10 ganglions droits et 10 gauches sans anomalies. Les suites postopératoires étaient simples. Puis la patiente était perdue de vue.

## Discussion

Les tumeurs mucineuses représentent 15 a 20% des tumeurs épithéliales de l’ovaire et seulement 2% des tumeurs de l’ovaire et grossesse. Pour la forme borderline, l’incidence annuelle est de 3,2 /100000 femmes dont l’âge varie entre 9 et 84 ans avec une moyenne de 35 ans, pouvant ainsi toucher des femmes en âge de procréer. Cependant l’association à la grossesse reste rare puisque la forme invasive ou borderline de l’ovaire ne concerne que 1/10 000 à 1/50 000 grossesse [1].

Les tumeurs mucineuses de l’ovaire représentent un véritable défi diagnostique pour le clinicien. Leur diagnostic ne se fait dans la plupart du temps, que tardivement du fait de leur symptomatologie frustre et non spécifique. Le diagnostic clinique est encore plus difficile au cours de la grossesse et la tumeur est le plus souvent retrouvée lors de l’échographie du premier trimestre [2] ou à l’échographie entre 22 et 32 SA. Cependant dans notre contexte comme c’est le cas de notre patiente, le diagnostic se fait le plus souvent tardivement lors d’une césarienne ou au stade de complications, vue la non généralisation de la consultation prénatale et des échographies systématiques.

En échographie pelvienne les tumeurs mucineuses sont de grandes tailles multiloculées réalisant le classique mais non spécifique aspect en nid d’abeille. Les lésions nodulaires, les végétations, Les cloisons épaisses, avec des raccordements géométriques et angulations brutales font évoquer la malignité [3]. La sensibilité de l’échographie vis-à-vis du diagnostic de malignité est de 93%. La TDM n’est supérieur pour le diagnostic de malignité que si elle met en évidence des signes d’extension extra pelvienne, de plus ses indications sont limitées au cours de la grossesse. L’IRM permet de différencier entre une tumeur bénigne et maligne avec une spécificité de 93% [4], et fait suspecter une tumeur borderline en présence de nombreux septas dans une tumeur multi kystique avec des végétations ou projections papillaires spécifiques aux tumeurs épithéliales [5]. Ainsi, L’IRM pelvienne n’a d’intérêt pendant la grossesse que devant une incertitude ou une insuffisance de l’échographie. Elle sera souvent d’autant plus intéressante que la grossesse est avancée et que la tumeur est volumineuse ce qui est le cas de notre patiente [6].

Une des particularités des tumeurs mucineuses est la difficulté de distinguer les tumeurs ovariennes primitives et métastatiques Les tumeurs mucineuses primitives devraient être beaucoup moins fréquentes que précédemment rapporté dans la littérature. Ceci peut être du a l’inclusion des tumeurs métastatiques dans la catégorie des tumeurs primitives. En effet, Selon une étude récente, la fréquence des adénocarcinomes mucineux primitifs n’est que de 2.4% de tous les carcinomes épithéliaux de l’ovaire. Des critères de distinction morphologiques et histologiques ont été élabores pour aider au diagnostic différentiel [7]. Autre particularité des tumeurs mucineuses est Le pseudomyxome péritonéal [8] (l’accumulation de mucine extracellulaire dans la cavité péritonéale). De plus, un conflit existe sur son origine puisque plusieurs études récentes s’appuyant sur des analyses immuno-histochimiques, génétiques et de biologie moléculaire ont démontré l’origine appendiculaire de la quasi-totalité des pseudomyxomes péritonéaux avec extension secondaire ovarienne éventuelle [8]. Et les seules tumeurs ovariennes primitives capables d’une authentique dissémination pseudomyxomateuse seraient les tératomes kystiques matures peut être par l’existence d’un contingent gastro-intestinal dans ces tumeurs embryonnaires [9]. Ainsi notre observation est un cas rare de pseudomyxome péritonéal d’origine ovarienne puisque l’étude histologique de l’appendice et de l’ovaire controlatéral est normale, ainsi que l’exploration du reste du tube digestif, ceci en plus de la présence d’un tératome mature. Par ailleurs, l’examen extemporané est peu fiable en cas de tumeur mucineuse particulièrement si elle est borderline ou de grande taille, et dépend de l’entraînement du pathologiste. Malgré cela, cet examen reste justifié [1].

Etant donné la rareté des cancers invasifs et des tumeurs borderlines de l’ovaire diagnostiqués au cours de la grossesse [1], les propositions de prise en charge thérapeutique dans cette situation doivent idéalement être structurées en Réunion de concertation pluridisciplinaire spécialisées (RCP)[1], cet avis faisant intervenir au minimum un chirurgien à compétence oncologique, un obstétricien, un oncologue médical et un pédiatre doit être fait chaque fois que possible très en amont de l’acte chirurgicale dès que le diagnostic est suspecté. De plus, toute décision engageant la grossesse (interruption) ou le foetus (chimiothérapie per gravidique ou accouchement prématuré) doit faire l’objet d’une évaluation bénéfice/risque dans la RCP spécialisée et doit être partagée avec la patiente et son conjoint [1].

Les tumeurs mucineuses borderlines confirmées peuvent bénéficier d’un traitement conservateur par annexectomie, appendicectomie, cytologie et exploration péritonéale avec biopsies de toute zone anormale. Ceci réduit le retentissement chirurgical potentiel sur la grossesse tout en conservant le pronostic de ces patientes. Ce diagnostic ne doit en aucun cas faire interrompre la grossesse [1–10]. Cependant la décision de notre équipe en concertation avec les oncologues était de faire un traitement radical vue que notre patiente était multipare, qu’elle refusait de se faire opérer une 2ème fois et que l’examen extemporané ne pouvait pas éliminer la présence de foyers d’invasion.

La chimiothérapie n’est indiquée qu’en présence des implants invasifs ou des lésions micro-invasives dans la tumeur primaire, et ceci étant donné leur nature relativement agressive [10].

## Conclusion

Etant donné qu’une part importante de la morbidité et de la mortalité associée aux tumeurs borderlines résulte du traitement plutôt que de la pathologie elle-même, nous incitons les gynéco-obstétriciens à adopter une attitude conservatrice chaque fois que la malignité n’est pas confirmée d’autant plus que la patiente est enceinte ou jeune. Par ailleurs dans le cas particulier des tumeurs mucineuses il faut toujours rechercher d’abord une origine digestive. En fin, en cas d’association tumeur ovarienne et grossesse, la décision thérapeutique ne se conçoit qu’en RCP - très en amont de l’acte chirurgical - en faisant participer le couple.
